# Cost-effectiveness analysis of anidulafungin for the treatment of candidaemia and other forms of invasive candidiasis

**DOI:** 10.1186/s12879-015-1143-1

**Published:** 2015-10-26

**Authors:** Georg Auzinger, E. Geoffrey Playford, Christopher N. Graham, Hediyyih N. Knox, David Weinstein, Michal Kantecki, Haran Schlamm, Claudie Charbonneau

**Affiliations:** King’s College Hospital, London, UK; Princess Alexandra Hospital, Brisbane, Australia; RTI Health Solutions, Research Triangle Park, Durham, NC USA; Pfizer International Operations, Paris, France; Pfizer Inc, New York, NY USA

**Keywords:** Anidulafungin, Candidaemia, Echinocandin, Invasive Candidiasis, Cost-Effectiveness

## Abstract

**Background:**

Candidaemia and other forms of invasive candidiasis (C/IC) in the intensive care unit are challenging conditions that are associated with high rates of mortality. New guidelines from the European Society for Clinical Microbiology and Infectious Diseases strongly recommend echinocandins for the first-line treatment of C/IC. Here, a cost-effectiveness model was developed from the United Kingdom perspective to examine the costs and outcomes of antifungal treatment for C/IC based on the European Society for Clinical Microbiology and Infectious Diseases guidelines.

**Methods:**

Costs and treatment outcomes with the echinocandin anidulafungin were compared with those for caspofungin, micafungin and fluconazole. The model included non-neutropenic patients aged ≥16 years with confirmed C/IC who were receiving intravenous first-line treatment. Patients were categorised as either a clinical success or failure (patients with persistent/breakthrough infection); successfully treated patients switched to oral therapy, while patients categorised as clinical failures switched to a different antifungal class. Other inputs were all-cause mortality at 6 weeks, costs of treatment-related adverse events and other medical resource utilisation costs. Resource use was derived from the published literature and from discussion with clinical experts. Drug-acquisition/administration costs were taken from standard United Kingdom costing sources.

**Results:**

The model indicated that first-line anidulafungin could be considered cost-effective versus fluconazole (incremental cost-effectiveness ratio £813 per life-year gained) for the treatment of C/IC. Anidulafungin was cost-saving versus caspofungin and micafungin due to lower total costs and a higher rate of survival combined with a higher probability of clinical success.

**Discussion:**

European Society for Clinical Microbiology and Infectious Diseases guidelines recommend echinocandins for the first-line treatment of C/IC; our model indicated that anidulafungin marries clinical effectiveness and cost-effectiveness.

**Conclusions:**

From the United Kingdom perspective, anidulafungin was cost-effective compared with fluconazole for the treatment of C/IC and was cost-saving versus the other echinocandins.

## Background

*Candida* species are the leading cause of invasive fungal disease worldwide and are one of the most common causes of hospital-acquired bloodstream infections [1], particularly in intensive care unit patients and very low birth weight infants [2]. Invasive candidiasis is a challenging condition that is associated with significant morbidity and mortality [1]. Although *C. albicans* is still the leading cause of invasive candidiasis in most clinical settings [3, 4], recently there has been a shift towards other *Candida* species, including *C. glabrata* and *C. krusei* [3, 5, 6]. This change has been attributed to the selection of less-sensitive *Candida* strains by the widespread use of fluconazole as a prophylactic and therapeutic agent [7, 8].

Due to the relatively high rate of infection by *Candida* species [9], the associated mortality [10], the high costs of hospitalisation [11], and the increasing prevalence of non-*albicans Candida* species [1], it is imperative that treatments that are both clinically and cost-effective are identified. The European Society for Clinical Microbiology and Infectious Diseases strongly recommends the use of the echinocandins anidulafungin, caspofungin and micafungin for the targeted first-line treatment of invasive candidiasis and suggests a downgrading of the conventional therapies, liposomal amphotericin B and fluconazole [12]. This recommendation is based on evidence indicating that echinocandins are highly active against a wide range of *Candida* species, that resistance is rare and that all agents are well tolerated with similar safety profiles and few drug–drug interactions [13]. Of the echinocandins, anidulafungin is the only drug in the class to have demonstrated superiority over fluconazole in the treatment of severely ill patients with invasive candidiasis [14, 15].

Health economic analyses are increasingly important in the clinical arena where decision-makers face growing pressure to optimise value and quality of care. Indeed, sources such as the Consensus Statement on the Role of Cost-Effectiveness Analysis in Health and Medicine recommend the use of cost-effectiveness analyses to assist decision-makers and the incorporation of economic data into treatment guidelines [16]. Factors contributing to the economic burden of invasive candidiasis include inpatient and outpatient costs, such as costs related to hospitalisation, increased length of stay in hospital, drug-acquisition costs and costs related to the treatment of drug-related adverse events [11].

The aim of this study was to examine the costs and outcomes associated with antifungal treatments for candidaemia (the most common manifestation of invasive candidiasis) and other forms of invasive candidiasis (collectively referred to as ‘C/IC’ throughout this article) from the United Kingdom hospital perspective using a decision analytic model based on European Clinical guidelines [12].

## Methods

### Study design overview

The economic analysis was performed from the perspective of the United Kingdom National Health Service and Personal and Social Services. The study population comprised non-neutropenic patients aged ≥16 years with confirmed C/IC receiving intravenous first-line treatment. As the study modelled data from the published literature, ethics approval and patient consent were not required.

**Fig. 1:**
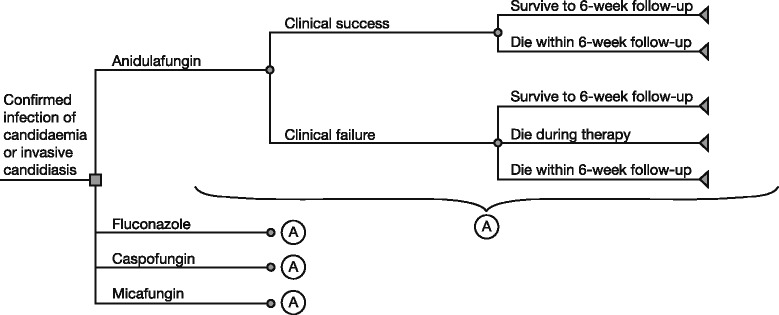
Model structure

A decision analytic model was constructed in Microsoft Excel to estimate the potential treatment costs of anidulafungin versus comparator agents (Fig. 1). A decision tree was deemed to be the most appropriate model to use in this analysis due to the short time horizons associated with the treatment of C/IC. The model time horizon was the 6-week inpatient follow-up period with an extrapolation to an average patient’s lifetime for those surviving the 6-week period. Extrapolation to a lifetime perspective was carried out using similar methods to those described by Neoh and colleagues [17] by applying a relative risk of death for a similar patient population (survivors of sepsis [18]) to the average life-expectancy of individuals aged 58 years (i.e. the average age of patients in the study by Reboli and colleagues [15]) in the United Kingdom. Life expectancy for survivors was 12.9 years. Life years were discounted at 3.5 % per annum. Each pathway in the model was defined by the probability of an event occurring and the costs associated with each clinical outcome.

The comparator agents were the other current European Society for Clinical Microbiology and Infectious Diseases-recommended first-line standards of care, the echinocandins caspofungin and micafungin [12], and (based on data from the trial by Reboli and colleagues) the generic and frequently used azole, fluconazole [15]. The second-line agents were liposomal amphotericin B for patients who received an echinocandin as first-line therapy, and anidulafungin, caspofungin or micafungin for patients who received fluconazole as first-line therapy.

Clinical efficacy (clinical success and failure) and mortality probabilities (Table 1) were extracted from a mixed-treatment comparison study of antifungal treatments for invasive *Candida* infections [19]. Hospital costs, drug doses and treatment-related adverse events were derived from a panel of clinical experts, the published literature, clinical trials and product labels. Antifungal drug-acquisition costs and administration costs were taken from standard United Kingdom costing sources [20].

**Table 1:** Clinical success and mortality [19]

Drug	Clinical success	Mortality
Anidulafungin	77.5 %	20.8 %
Fluconazole	63.0 %	28.4 %
Caspofungin	76.1 %	33.8 %
Micafungin	76.0 %	39.2 %

The outcome measures for the model were the total costs, antifungal drug costs and other medical costs associated with each treatment in 2012 pounds sterling (£). Incremental cost-effectiveness ratios for the incremental cost per life-year gained (i.e. the cost associated with achieving one additional year of patient survival) with each antifungal treatment were calculated using the formula:

$$ \mathrm{ICER}=\left(\mathrm{C}1-\mathrm{C}2\right)/\left(\mathrm{E}1-\mathrm{E}2\right), $$

where C1 and E1 were the cost and efficacy of anidulafungin and C2 and E2 were the cost and efficacy of the comparator agent.

An intervention was deemed to be cost-effective when its incremental cost-effectiveness ratio compared with the comparator agent fell below a certain pre-defined threshold. An intervention was deemed to be dominant, or cost-saving, when it demonstrated both better effectiveness and lower costs.

### Key model assumptions and inputs

#### Patients

The average weight of patients in the anidulafungin arm of the Reboli and colleagues [15] trial was 76.4 kg (± standard deviation 25.5 kg). Assuming that weight followed a normal distribution, it was estimated that 55.6 % of patients weighed ≤80 kg and 44.4 % of patients weighed >80 kg (relevant to caspofungin dosing) and that 7.7 % of patients weighed ≤40 kg and 92.3 % of patients weighed >40 kg (relevant to micafungin and voriconazole dosing).

#### Treatment pathway

Following usual treatment patterns, patients who achieved clinical success received intravenous antifungal treatment for 10 days (3 days prior to clearance plus one additional week) followed by 7 days of oral treatment with fluconazole. All patients received an intravenous loading dose followed by intravenous maintenance dosing of antifungal therapy. No patients had their drug dose titrated and it was assumed that there was no vial wastage in drug usage.

Clearance of C/IC in patients who were successfully treated (i.e. achieved persistent negative blood cultures) was assumed to take 3 days and, therefore, clinical determination of first-line treatment failure was made on Day 3. In accordance with the Infectious Diseases Society of America and European Society for Clinical Microbiology and Infectious Diseases guidelines [12, 21], patients remained on antifungal therapy for 14 days after their first negative blood culture (7 days of intravenous therapy plus 7 days of oral fluconazole therapy).

If an infection persisted or breakthrough infection occurred (treatment failure) then a switch in antifungal agents was assumed to take place. Patients switched to the next level of recommended therapy available after the first-line failure [12]. For patients who received an echinocandin as first-line treatment, second-line treatment was liposomal amphotericin B (B-level [moderate] recommendation). After failure of the azole fluconazole as first-line treatment, one-third of patients were treated with anidulafungin, one-third received caspofungin and one-third received micafungin (A-level [strong] recommendation). The duration of second-line treatment was equivalent to the assumptions made for first-line treatment. It was assumed that only two lines of treatment were required to clear the infection.

#### Resource use

Resource use was derived from United States database analysis of C/IC treatment clinical success and failure (Premiere Database, Pfizer, Data on File). Data were validated by clinical experts and were assumed to reflect current practice in the European Union and the United Kingdom. Length of hospitalisation differed between patients whose treatment was successful and patients whose treatment failed (Table 2). Most resource costs associated with antifungal treatment were assumed to be related to the length of bed stay, whereas other costs, such as laboratory costs, were assumed to be minimal and, therefore, were not modelled [22].

**Table 2:** Hospitalisation length of stay and costs

	Intensive care unit		Other hospital	
Outcome in hospital setting	Length of stay^a^ (days)	Cost per day^b^ (£)	Length of stay^a^ (days)	Cost per day^b^ (£)
Clinical success, survive	11.5	1.528	13.0	242
Clinical success, die	12.8		12.8	
Clinical failure, survive	19.0		23.3	
Clinical failure, die	20.3		19.5	

^a^Pfizer, Data on File; ^b^Sidhu et al. [22]

#### Adverse events

As the goal of the cost-effectiveness analysis was to assess economic impact, adverse events that did not require medication or resource use were not included.

Nephrotoxicity is associated with the polyene class of antifungal therapies, which includes liposomal amphotericin B [12, 23]. Although other toxicities may occur with antifungal agents, they were assumed to be neither costly nor associated with a treatment switch. The probabilities of experiencing nephrotoxicity with liposomal amphotericin B were extracted from the AmbiSome product label [24]. Nephrotoxicity probabilities for the echinocandins were extracted from the published data [19], which reported that, compared with liposomal amphotericin B, echinocandins and azoles have relative risks of nephrotoxicity of 0.31 (95 % confidence interval 0.17–0.57) and 0.22 (95 % confidence interval 0.15–0.32), respectively. Costs for nephrotoxicity adverse events were applied to the decision tree by multiplying the incidence of each adverse event by the average cost of treating and managing the adverse event. Costs for treating and managing each adverse event were estimated to be £1693.44 based on approximately 7 days of additional hospital stay [25]. Costs were estimated as the average of non-intensive care unit hospital costs per day for 7 days [25].

#### Mortality

All-cause mortality data were taken from estimates in the published literature for overall treatment [19]. To accurately project drug-acquisition costs for the patients that died during therapy and those that completed therapy, the proportion of patients who died following treatment failure was determined using data derived from the study by Reboli and colleagues (Pfizer, Data on File). Among the modified intent-to-treat population, 34 % of anidulafungin-treated patients and 46 % of fluconazole-treated patients died during therapy (Pfizer, Data on File). The average of these two values (41 %) was used in the model for all agents. The average time to death in patients who died during the 6-week study period was assumed to be approximately 22.8 days, based on a mean time to death of 22.9 days for anidulafungin and 22.6 days for fluconazole (Pfizer, Data on File). Patients who experienced clinical failure and died were assumed to die within 7 days of commencing treatment.

### Sensitivity analysis

To examine the robustness of the model to alternative input parameters and assumptions, we conducted a one-way sensitivity analysis. Here, individual parameters were varied independently with the usual convention being that both a value less than and a value higher than the base-case input parameter were tested. We summarised the results of the one-way sensitivity analysis by examining the effect of changing parameter values or assumptions on the total cost of treatment. Specifically, we looked at the total incremental costs of caspofungin and micafungin compared with anidulafungin, where positive costs indicated that caspofungin and micafungin were more expensive in the scenario than anidulafungin.

## Results

### Base-case analysis

**Fig. 2:**
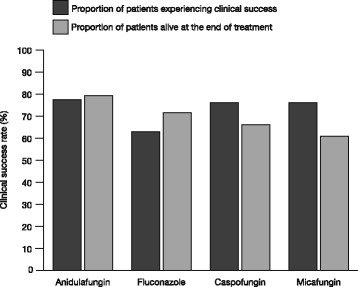
Treatment outcomes for each antifungal treatment

The proportion of patients who achieved clinical success and the proportion of patients who were alive at the end of treatment were both >75 % for anidulafungin (Fig. 2). Higher clinical success rates and survival rates were reported for anidulafungin compared with caspofungin, fluconazole and micafungin (Fig. 2).

**Fig. 3:**
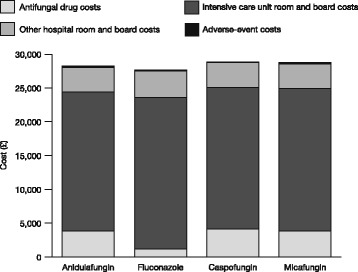
Treatment costs associated with each antifungal agent

Drug costs were lower for generic fluconazole compared with anidulafungin, caspofungin and micafungin. Intensive care unit costs made up the greatest proportion of costs across all of the assessed agents (Fig. 3). Other hospital room and board costs and adverse events were comparable across all of the comparator agents (other hospital room and board costs included costs other than antifungal drug costs that were incurred in addition to the intensive care unit costs, and included room and board costs on a general hospital ward).

In terms of the incremental costs per life-year gained, treatment with first-line anidulafungin could be considered cost-effective compared with fluconazole due to the very low incremental cost-effectiveness ratio, and was cost-saving compared with caspofungin and micafungin (Table 3). The cost savings associated with anidulafungin compared with caspofungin and micafungin were attributable to lower intensive care unit costs, and a higher rate of survival combined with a higher probability of clinical success.

**Table 3:** Costs, life-years and incremental cost per life-year gained

First-line treatment	Cost per loading dose (£)	Cost per maintenance dose (£)		Total costs (£)	Incremental costs (£)	Total no. life years	Incremental no. life years	Incremental cost-effectiveness ratio
	IV	IV	Oral					
Anidulafungin	600.0	300.0	-	28,216	–	7.23	–	–
Fluconazole	7.8	7.8	1.4	27,646	−570	6.52	−0.70	813
Caspofungin	416.8	367.2	-	28,905	689	6.03	−1.19	Dominated^a^
Micafungin	341.0	341.0	-	28,721	504	5.55	−1.68	Dominated^a^

The incremental cost-effectiveness ratio calculated was the incremental cost per life year gained. All incremental cost-effectiveness ratios were calculated as anidulafungin vs comparator

^a^First-line treatment with anidulafungin was less costly and more effective than the comparator, thus first-line treatment with anidulafungin dominated the comparator

### One-way sensitivity analysis

Using data direct from the study by Reboli and colleagues in a sensitivity analysis, anidulafungin remained cost-effective when compared with fluconazole (not shown) [15]. With regards to the echinocandins, we investigated alternative assumptions and parameters in a one-way sensitivity analysis. Table 4 shows the incremental total costs of caspofungin and micafungin compared with anidulafungin for each variation of input parameter. Anidulafungin remained a cost-saving option compared with the other echinocandins across most of the alternative assumptions.

**Table 4:** Incremental total costs of caspofungin and micafungin compared with anidulafungin for each variation of input parameter in a one-way sensitivity analysis

Parameter	Basecase	Change in input lower than base case			Change in input higher than base case		
		Input	Incremental costs versus anidulafungin^a^		Input	Incremental costs versus anidulafungin^a^	
			Caspofungin	Micafungin		Caspofungin	Micafungin
Anidulafungin drug-acquisition costs (£)	£300 per vial	−20 %	1252	1068	+20 %	126	−59
Mortality	[19]	Assumed equivalent	535	287	NA	NA	NA
Clinical success and mortality	[19]	Assumed equivalent	316	47	NA	NA	NA
Clinical success, survive, ICU LOS (days)	11.5	7	1446	1544	14	263	−80
Clinical success, die, ICU LOS (days)	12.8	7	−166	−705	14	868	757
Clinical failure, survive, ICU LOS (days)	19.0	10	968	949	30	349	−37
Clinical failure, die, ICU LOS (days)	20.3	10	151	−241	30	1194	1205
ICU cost per day (£)	1528	1000	544	318	2000	819	672
Time to clinical failure (treatment switch) (days)	3	1	654	482	5	724	527
Clinical failure and die, time to death (days)	7	3	717	543	10	668	475
Clinical success, IV treatment duration (days)	10	7	536	415	14	892	624

*ICU* intensive care unit; *IV* intravenous; *LOS* length of stay; *NA* not available

^a^Positive costs indicate that the total cost of the comparator regimen is more expensive than anidulafungin; negative costs indicate that the comparator regimen is less expensive than anidulafungin

The one-way sensitivity analysis indicated that it would take an increase of 20 % in anidulafungin drug-acquisition costs for micafungin to be less costly than anidulafungin. Also, assuming a class effect for mortality and survival did not change the model conclusions as anidulafungin remained cost-saving. Shortening the length of stay for patients who died allowed micafungin and caspofungin to become less costly than anidulafungin, owing to the greater clinical success of anidulafungin (i.e. more micafungin and caspofungin patients were projected to fail and, thus, with shorter length of stay for failure patients, less costs were accrued).

## Discussion

The relative cost of antifungal therapies has become an increasingly important issue in recent years due to growing concerns about the rising costs of healthcare and the lack of data demonstrating the superiority of one agent over another [26]. Pharmacoeconomic analyses can play an important and useful role in the allocation of healthcare resources, and integrate the efficacy and safety data obtained from clinical trials, and healthcare resource use and quality-of-life information from the literature, expert opinion and analysis of databases [27].

The pharmacoeconomic evaluation described in this paper applies to the treatment of C/IC in non-neutropenic patients from the United Kingdom perspective. Our model demonstrated that anidulafungin could be considered cost-effective compared with fluconazole in terms of life years gained with total costs below the generally accepted United Kingdom cost-effectiveness threshold (often referred to as society’s willingness to pay for an additional unit of health gain, the ‘quality-adjusted life year’) of £20,000-£30,000 [28]. Furthermore, the results showed that the agents within the echinocandin class had similar costs and efficacy. Within the echinocandin class, the model estimated that anidulafungin was cost-saving compared with caspofungin and micafungin due to higher efficacy (clinical success and survival, as estimated using data from Mills and colleagues [19]) and lower total costs. Examining the costs further, the clinical efficacy benefit for anidulafungin potentially drove savings in intensive care unit days. Additionally, drug-acquisition costs were lower for anidulafungin.

Overall, the clinical effectiveness and cost-effectiveness of anidulafungin indicate that it is a sensible option for the treatment of C/IC in a cost-constrained economic environment, such as the United Kingdom National Health Service. Although our results are only valid for the United Kingdom due to the local nature of costs, our findings are consistent with previous cost-effectiveness analyses of anidulafungin for the treatment of C/IC. In a pharmacoeconomic analysis comparing the cost-effectiveness of anidulafungin and fluconazole for the treatment of C/IC in Spain, Grau and colleagues reported that overall treatment costs with anidulafungin were lower compared with fluconazole (€37,240 versus €37,327, respectively) even though its drug-acquisition costs were more than two-fold greater (€5,780 versus €2,082, respectively) [29]. Furthermore, anidulafungin treatment resulted in a higher clinical success rate compared with fluconazole (74 % versus 57 %, respectively) and was associated with an incremental cost-effectiveness ratio of -€505 [29]. Reboli and colleagues performed an economic evaluation of data from their randomised controlled trial of anidulafungin versus fluconazole which reported a significantly higher overall success rate for anidulafungin in the protocol-defined primary endpoint [15]. The results of the economic analysis demonstrated that anidulafungin was a cost-effective alternative to fluconazole with cost savings of $2,223 (expert assessment) and $2,681 (regression analysis), respectively, for anidulafungin [30]. In a pharmacoeconomic evaluation of anidulafungin, caspofungin and micafungin from the Spanish hospital perspective, Garcia and colleagues reported that anidulafungin was cost-saving with a lower drug-acquisition cost (€6,000) per episode than caspofungin (€4,665 to €7,991) and micafungin (€6,000 to €10,714) [31].

Some limitations of the study need to be taken into account when interpreting these results. First, the use of efficacy data from a mixed-treatment comparison of randomised clinical trials [19] may limit the ability to generalise the findings of this analysis to a broader population, and more up-to-date clinical data may now be available. Studies included in the meta-analysis had differing definitions of response and timing of assessments and may have included selected populations that had effects on fungal-attributable and all-cause mortality [19]. To account for such differences, the authors conducted several sensitivity analyses, including analyses around fungal-attributable mortality, and concluded that their results were robust [19]. In our sensitivity analyses, we held mortality and survival probabilities equivalent for all echinocandins and found anidulafungin remained cost-saving in these scenarios due to differences in drug-acquisition costs. Furthermore, the assumption that second-line treatment had efficacy of 100 % is a simplification of reality. However, this assumption was discussed with clinical experts and was considered acceptable. Finally, data from the United States were used to estimate hospital length of stay; however, total hospitalisation costs using these data were similar to those reported in a United Kingdom analysis of caspofungin and micafungin [22]. We attempted to ascertain the impact of parameter uncertainty and model assumptions via alternatives in a one-way sensitivity analysis. In most alternative scenarios conducted, anidulafungin remained a cost-saving treatment compared with caspofungin and micafungin.

Further analyses using the model reported here will be used to compare the cost-effectiveness of anidulafungin with conventional amphotericin B and voriconazole, which are not commonly prescribed in the United Kingdom but may be applicable to other locales. In addition, future studies using observational real-world data are required to confirm the findings reported here for patients with C/IC in the United Kingdom.

## Conclusions

In conclusion, the results of this study show that, from the United Kingdom perspective, anidulafungin was cost-effective compared with fluconazole, and cost-saving compared with the other available echinocandins caspofungin and micafungin, for the treatment of C/IC.

## Abbreviations

C/IC: Candidaemia and other forms of invasive candidiasis
ESCMID: European Society for Clinical Microbiology and Infectious Diseases
ICU: Intensive care unit
IV: intravenous
LOS: Length of stay
NA: Not available
UK: United Kingdom
